# Supplementary material for: Pilot Investigation on Markers of Bone Metabolism, Angiogenesis, and Neuroendocrine Activity as Potential Predictors of Survival of Metastatic Prostate Cancer Patients with Bone Metastases
Source: Int J Mol Sci. 2025 May 13;26(10):4669. doi: 10.3390/ijms26104669 (PMC12111599; doi:10.3390/ijms26104669)
Supplement: Supplementary file 1 [file ijms-26-04669-s001.zip › ijms-3540070-supplementary.pdf]

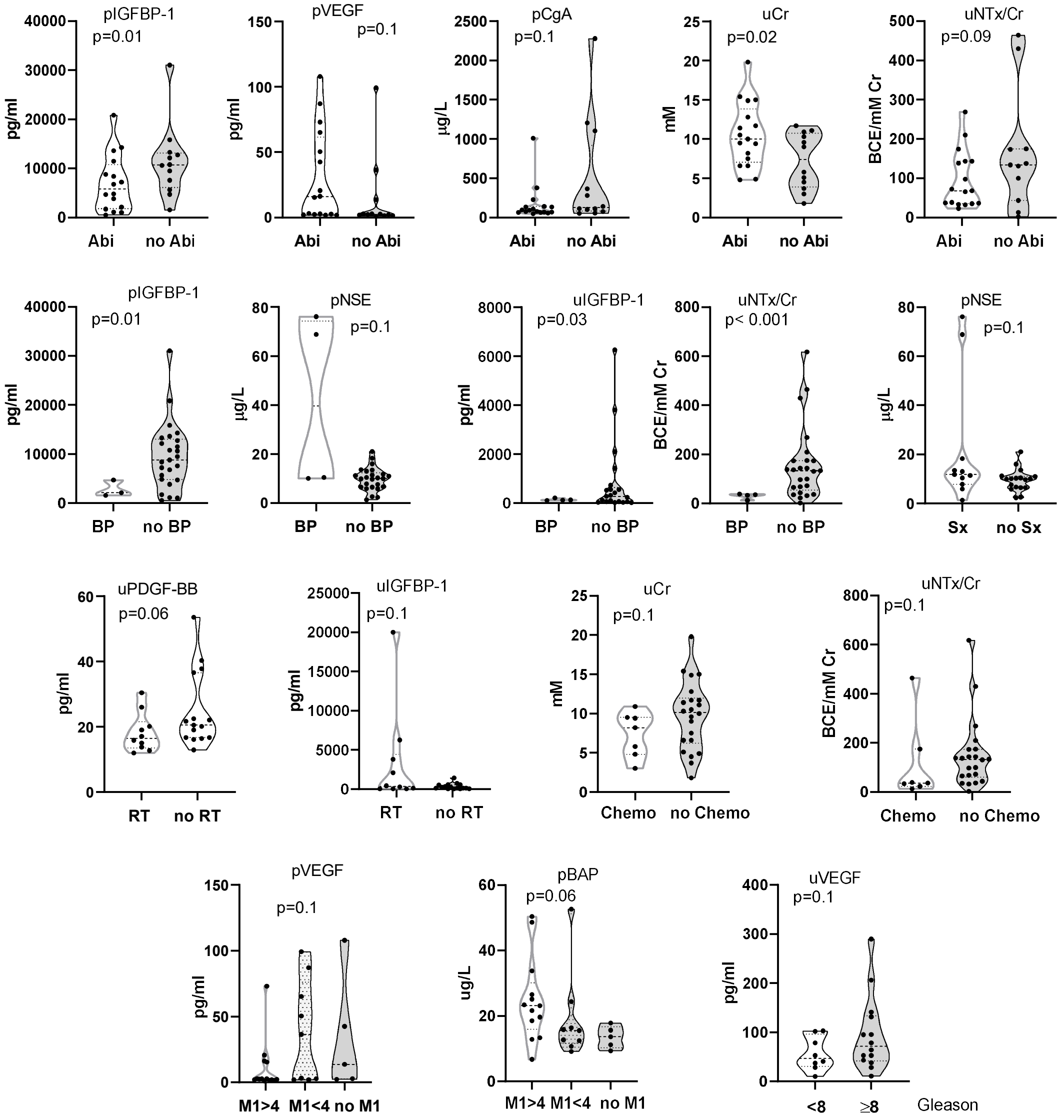

Figure S1. Graphical representation of the peripheral blood and urinary levels of molecules from Table 2.

Table S1. Number of patients from C1, C2 and C3 included in each subgroup defined by the presence or absence of treatments or clinical characteristics.

	C1 (n)	C2 (n)	C3 (n)
**Abi**			
si	5	8	3
no	3	4	5
**BP**			
si	0	2	1
no	8	10	7
**Sx**			
si	4	6	1
no	4	6	7
**RT**			
si	3	5	4
no	5	7	4
**Chemo**			
si	1	3	2
no	7	9	6
**gleason >=8**			
si	6	6	4
no	2	5	1
**M1 at t0**			
>4	1	7	6
<4	5	3	1
no	2	2	1

For each treatment group (Abiraterone + prednisone [Abi], bisphosphonates [BPs], surgery [Sx], radiotherapy [RT], and chemotherapy [Chemo]), we indicated the number of patients who received the treatment ("yes") and those who did not ("no"). Similarly, it was done for Gleason score at diagnosis ≥ 8 (Gleason ≥ 8*). For bone metastasis at baseline (M1 at t0) we indicated the number of patients with more than 4 bone metastasis (>4), less than 4 bone metastasis (<4), or who did not presented bone metastasis.

Table S2. Summary of treatment duration at baseline (t0).

	Time at t0		Time at t0
ADT	50.7 (1-200) months	Abi	221 (6-1800) days
iADT	96.2 (12-168) months	BPs	12.6 (2.5-39) months
Sx	162.5 (24-276) months	Dx	9(1-23) years
RT	105 (2-228) months	M1	4.1 (0.083-19) years
QT	36.7 (0.6-72) months		

Data are presented as mean (range). ADT: Androgen deprivation therapy; iADT: Intermittent androgen deprivation therapy; Sx: Surgery; RT: Radiotherapy; QT: Chemotherapy; Abi: Abiraterone + prednisone; BPs: Bisphosphonates; Dx: diagnòstic; M1: metastases. For Sx, M1, and Dx, the table indicates the time elapsed until t0. For iADT, RT, QT, and Abi, the reported duration represents the time from treatment initiation to t0 but does not necessarily imply continuous treatment throughout this period.
